# DOES THE IMPACT OF COGNITIVE INTERVENTION ON MOBILITY VARY BY CHRONIC DISEASE?

**DOI:** 10.1093/geroni/igae098.3045

**Published:** 2024-12-31

**Authors:** Deborah Dadson, Karlene Ball

**Affiliations:** University of Alabama at Birmingham, Birmingham, Alabama, United States; University of Alabama at Birmingham, Birmingham, Alabama, United States

## Abstract

Mobility loss has been associated with chronic diseases, sedentary behavior, and fear of falls among older adults with approximately one-third of 70-year-olds and 80-year-olds reporting restrictions on mobility. Even though physical activity and exercise have been shown to enhance mobility and improve activities of daily living, only a few older adults are able to exercise due to several limiting factors. Cognitive training has been shown to enhance cognition, gait and motor functions, and consequently mobility among older adults. As such, this study aimed to examine how the impact of cognitive interventions on life space mobility varies by chronic disease. Using data from the Advanced Cognitive Training for Independent and Vital Elderly (ACTIVE) Clinical Trial, a sample of 2802 community dwelling older adults who were 65 years and older were examined using Multiple Linear Regression with interaction. Analyses included predicting life space mobility from the cognitive intervention trainings (reasoning, memory, and speed of processing), with gender, age, race, and mobility at baseline as covariates. Diabetes, cancer, and heart disease were entered into the model as moderators. Results indicated that speed of processing training, years of education and heart disease significantly predict Life Space Mobility (all p <.05). Reasoning training, memory training, gender, age, race, diabetes, and cancer were not uniquely associated with Life Space Mobility. These findings highlight the need to consider cognitive interventions, specifically speed of processing to ensure functional performance in everyday activities of older adults. Keywords: Mobility, cognitive intervention, chronic diseases, ACTIVE Clinical Trial

